# Chylothorax as an Initial Presentation of Waldenström Macroglobulinemia: Report of Two Cases

**DOI:** 10.1155/crh/7430484

**Published:** 2026-09-18

**Authors:** Yara Hamadah, Wan Ying Tan, Ursula M. A. de Matos, Ritika Vankina, Kapil Meleveedu, Swarup Kumar

**Affiliations:** ^1^ Department of Internal Medicine, University of Connecticut School of Medicine, Farmington, Connecticut, USA, uchc.edu; ^2^ Department of Hematology and Oncology, Carole and Ray Neag Comprehensive Cancer Center, UConn Health, Farmington, Connecticut, USA, uconnhealth.com

**Keywords:** bendamustine–rituximab, BTK inhibitor, case report, chylothorax, lymphoplasmacytic lymphoma, MYD88, pleural effusion, Waldenström macroglobulinemia

## Abstract

**Background:**

Chylothorax is an uncommon form of pleural effusion defined by the pathological accumulation of chyle within the pleural space. Although thoracic duct injury constitutes the most frequent cause, nontraumatic causes, including malignancies and lymphoproliferative disorders, merit consideration. Waldenström macroglobulinemia (WM), a lymphoplasmacytic lymphoma (LPL) characterized by bone marrow involvement and an immunoglobulin M (IgM) monoclonal gammopathy, rarely presents with pulmonary complications, and its association with chylothorax has been described in only a limited number of reports.

**Case Presentation:**

We describe two patients who presented with right‐sided chylothorax as the initial manifestation of WM. Both patients underwent comprehensive diagnostic evaluation confirming LPL and received multimodal management comprising therapeutic pleural interventions and WM‐directed systemic therapy.

**Conclusion:**

Chylothorax is a rare manifestation of WM and may represent the initial presentation of the disease. Early recognition, comprehensive diagnostic evaluation, and initiation of appropriate disease‐directed therapy are essential for symptom control and management. Further studies are needed to better characterize the mechanisms, optimal treatment strategies, and long‐term outcomes of WM‐associated chylothorax.

## 1. Introduction

Chylothorax refers to the pathologic accumulation of chyle within the pleural space. The condition most commonly results from injury to the thoracic duct, typically of traumatic or iatrogenic origin; however, nontraumatic causes must also be considered. Chylothorax is diagnostically distinguished from other pleural effusions by pleural fluid triglyceride (TG) levels exceeding 110 mg/dL, the presence of chylomicrons, a relatively low cholesterol level, and an elevated lymphocyte count [1]. Clinically significant chylothoraces may result in hypovolemia, malnutrition, and immunosuppression due to the loss of essential proteins, immunoglobulins, lipids, vitamins, and water.

Malignancies, including lymphoproliferative disorders, have rarely been reported as causes of chylothorax. Malignant lymphadenopathy can lead to chylothorax through compression of the thoracic duct and regional lymphatic channels by enlarged lymph nodes, impeding lymphatic drainage from the periphery of the lung parenchyma and pleural space. The anatomic level of thoracic duct obstruction determines laterality: obstruction below the level of thoracic vertebra T5 generally results in right‐sided effusions, while obstruction above T5 results in a left‐sided chylothorax [2].

Waldenström macroglobulinemia (WM) is a clinicopathologic entity defined by the presence of lymphoplasmacytic lymphoma (LPL) in the bone marrow with an associated immunoglobulin M (IgM) monoclonal gammopathy [1]. Recent advances in the understanding of WM pathogenesis have highlighted the role of recurrent molecular alterations, particularly MYD88 L265P mutations, which activate nuclear factor‐κB (NF‐κB) signaling and promote malignant B‐cell survival, and CXCR4 mutations, which modulate tumor cell trafficking and influence response to Bruton’s tyrosine kinase (BTK) inhibitors. Additional genetic abnormalities, including TP53 alterations, have been associated with disease progression, treatment resistance, and inferior clinical outcomes, emphasizing the growing importance of molecular profiling in WM management [3].

The most common presenting features of WM include anemia, constitutional symptoms, symptomatic lymphadenopathy, and hyperviscosity syndrome, with at least 25% of patients being asymptomatic at diagnosis. Pulmonary compromise is uncommon, and the association of chylothorax and WM has been reported only a limited number of times in the literature [4]. We present a report of two cases identified at our institution to further illustrate this rare association and to review available management strategies.

## 2. Case Presentation

The baseline clinical and laboratory characteristics of both patients are summarized in Table 1. A timeline of diagnostic and therapeutic milestones is presented in Table 2.

**TABLE 1:** Baseline clinical and laboratory characteristics of patients presenting with chylothorax associated with Waldenström macroglobulinemia.

Characteristic	Patient A	Patient B
Patient information		
Age (years)	70	69
Sex	Male	Male
Relevant comorbidities	Type 1 DM, HTN, CAD, schizophrenia	Stage IV rectal adenocarcinoma (on surveillance), HTN, CKD stage 3^a^
Date and site of presentation	September 2023; UConn John Dempsey Hospital	December 2024; UConn John Dempsey Hospital
Pleural fluid analysis		
Pleural effusion laterality	Right	Right
Pleural fluid TG level (mg/dL)	408	261
Pleural fluid cytology	Negative for malignant cells	Consistent with B‐cell lymphoma involvement
Pleural fluid flow cytometry	Negative for malignant cells	Clonal B‐cell population: CD19+, CD20+, CD5 equivocal
Imaging		
Imaging modality	CT abdomen and pelvis	CT (surveillance imaging)
Lymphadenopathy site (s)	Retroperitoneal (nonspecific)	Mesenteric, portacaval, inguinal
Tissue diagnosis		
Biopsy type	Bone marrow biopsy	Excisional lymph node biopsy (left inguinal) + bone marrow aspirate
Histopathologic diagnosis	Kappa‐restricted B‐cell lymphoma (LPL)	Atypical B‐cell lymphoid proliferation consistent with B‐cell lymphoma (LPL)
Immunophenotype	CD19+, CD20+, CD5−, CD10−, CD25−	CD20+, CD5−, CD10−, CD23+; kappa‐restricted; Ki‐67 20%–30%
Bone marrow involvement	60%–70%; cellularity 99%; decreased trilineage hematopoiesis; no excess blasts	Atypical kappa‐restricted B‐cell population consistent with mature B‐cell lymphoma (% not reported)
Laboratory findings at diagnosis		
Serum IgM level (mg/dL)	5207	297
Monoclonal protein type	IgM kappa	IgM kappa
M‐Component (g/dL)	Not available	0.2
Free kappa light chains (mg/L)	Not available	68.83
Free lambda light chains (mg/L)	Not available	14.75
Kappa/lambda ratio	Not available	4.67
Platelet count (× 10^3^/μL)	Nadir 60 (decreased from 200)	Thrombocytopenia (value not reported)
Anemia at presentation	*Not available*	Mild anemia (hemoglobin not reported)
Molecular and cytogenetic testing		
MYD88 L265P mutation	Detected (VAF 2.0%; by NGS)	Not detected
CXCR4 mutation status	Not available	Not available
Cytogenetics/karyotype	Abnormal; deletion of chromosome 6q	Normal karyotype
Treatment and outcomes		
Initial WM‐directed therapy	BR (rituximab omitted cycle 1 due to IgM flare risk; baseline IgM > 4000 mg/dL)	Single‐agent rituximab (initial); subsequently escalated to BR given CKD and baseline cytopenias
Additional pleural intervention	Chest tube placement (prior to WM diagnosis); 1 recurrence after treatment initiation; resolved after 2 BR cycles	VATS pleurodesis (January 2025) for refractory recurrent chylothorax despite rituximab monotherapy
Subsequent therapy	Zanubrutinib 160 mg daily (comorbidities and chemotherapy intolerance)	Rituximab + acalabrutinib (second‐line; initiated May 2026 for confirmed disease recurrence)
Best IWWM response	VGPR (at 10 months)	VGPR (at 6 months on BR)
Follow‐up status	Ongoing response on zanubrutinib; no recurrent chylothorax as of June 2026	Disease recurrence confirmed May 2026; progressive lymphadenopathy and recurrent pleural effusion; second‐line therapy initiated

Abbreviations: BR, bendamustine–rituximab; CAD, coronary artery disease; CKD, chronic kidney disease; CT, computed tomography; DM, diabetes mellitus; HTN, hypertension; IgM, immunoglobulin M; IWWM, International Workshop on Waldenström Macroglobulinemia; LPL, lymphoplasmacytic lymphoma; NGS, next‐generation sequencing; TG, triglyceride; VAF, variant allele frequency; VATS, video‐assisted thoracoscopic surgery; VGPR, very good partial response; WM, Waldenström macroglobulinemia.

^a^CKD attributed to recurrent acute kidney injury in the setting of prior metastatic colorectal cancer treatment, multiple chemotherapy exposures, and prior left renal infarction resulting in an atrophic left kidney with a functionally solitary right kidney.

**TABLE 2:** Timeline of diagnostic and therapeutic milestones.

Time point	Patient A	Patient B
Month 0 (initial presentation/prediagnosis)	**August 2023:** hospitalized for NSTEMI. Incidental right‐sided pleural effusion identified; chest tube placed. Pleural fluid: TG 408 mg/dL, RBC 3000/mm^3^. Cytology and flow cytometry negative for malignancy. CT abdomen/pelvis: nonspecific retroperitoneal lymphadenopathy	**November 1, 2024:** excisional biopsy of left inguinal lymph node: atypical B‐cell lymphoid proliferation (CD20+, CD5−, CD10−, CD23+; kappa‐restricted; Ki‐67 20%–30%). Surveillance CT: progressive mesenteric, portacaval, and inguinal lymphadenopathy
Month 1 (chylothorax identification)	**September 2023:** re‐hospitalized for persistent dyspnea and productive cough. Re‐accumulation of right‐sided chylothorax confirmed. Worsening thrombocytopenia: platelets 200 ⟶ 60 × 10^3^/μL	**December 2024:** presents with progressive dyspnea. Large right‐sided pleural effusion confirmed as chylothorax (TG 261 mg/dL). Pleural fluid cytology and flow cytometry: clonal B‐cell lymphoma (CD19+, CD20+, CD5 equivocal). Mild anemia and thrombocytopenia noted
Months 1–2 (diagnostic workup and WM diagnosis)	Bone marrow biopsy: kappa‐restricted B‐cell lymphoma (LPL); 60%–70% involvement; cellularity 99%; decreased trilineage hematopoiesis; no excess blasts. Serum IgM 5207 mg/dL. Immunophenotype: CD19+/CD20+/CD5−/CD10−/CD25−. **Molecular testing:** MYD88 L265P detected (VAF 2.0%; NGS). Cytogenetics: deletion of Chromosome 6q. **WM diagnosis confirmed**	Bone marrow aspirate: atypical kappa‐restricted B‐cell population. Serum IgM 297 mg/dL; M‐component 0.2 g/dL; kappa/lambda ratio 4.67. **Molecular testing:** MYD88 L265P not detected. Cytogenetics: normal karyotype. **WM diagnosis confirmed**
Month ∼2 (initiation of WM‐directed therapy)	BR chemoimmunotherapy initiated. Rituximab omitted from Cycle 1 (baseline IgM > 4000 mg/dL; IgM flare risk). One additional episode of chylothorax after treatment initiation	Single‐agent rituximab initiated given CKD and baseline cytopenias. Recurrent symptomatic chylothorax persists despite monotherapy
Months ∼3–4 (pleural intervention/treatment escalation)	Pleural fluid re‐accumulation resolves after 2 cycles of BR. No further thoracenteses required	**January 2025:** VATS pleurodesis performed for refractory recurrent chylothorax. Bendamustine added to rituximab (BR regimen initiated)
Month ∼6 (treatment response/therapy change)	Transitioned from BR to zanubrutinib 160 mg daily due to comorbidities and chemotherapy intolerance	VGPR achieved as per IWWM criteria after BR therapy (4 cycles). Improvement in respiratory symptoms and functional status. No further therapeutic thoracenteses required during the initial response period
Month 10 (best response — Patient A only)	VGPR achieved per IWWM criteria	—
Most recent follow‐up	**June 2026:** ongoing response on zanubrutinib 160 mg daily. No treatment‐related cytopenias. No recurrent chylothorax. Preserved functional status; continued outpatient hematology follow‐up	**May 2026:** hospitalized with recurrent progressive dyspnea and pleuritic chest discomfort. Imaging: persistent loculated right pleural effusion with pleural calcifications; progressive mediastinal, hilar, axillary, mesenteric, inguinal, and retroperitoneal lymphadenopathy; new soft tissue lesions. Recurrent B‐cell lymphoma confirmed. Second‐line therapy initiated: rituximab + acalabrutinib

*Note:* Time points are relative to the index chylothorax hospitalization for Patient A (September 2023) and the index chylothorax presentation for Patient B (December 2024). Absolute dates are provided where available.

Abbreviations: BR, bendamustine–rituximab; CT, computed tomography; IgM, immunoglobulin M; IWWM, International Workshop on Waldenström Macroglobulinemia; LPL, lymphoplasmacytic lymphoma; NGS, next‐generation sequencing; NSTEMI, non‐ST elevation myocardial infarction; RBC, red blood cell; TG, triglyceride; VAF, variant allele frequency; VATS, video‐assisted thoracoscopic surgery; VGPR, very good partial response; WM, Waldenström macroglobulinemia.

### 2.1. Patient A

#### 2.1.1. Patient Information

A 70‐year‐old male with a history of Type 1 diabetes mellitus (DM), hypertension (HTN), coronary artery disease (CAD), and schizophrenia presented to UConn John Dempsey Hospital in September 2023 with recurrent right‐sided chylothorax.

#### 2.1.2. Clinical Findings

One month prior to presentation, the patient had been hospitalized for a non‐ST elevation myocardial infarction (NSTEMI), during which he was incidentally found to have a right‐sided pleural effusion requiring chest tube placement. Initial pleural fluid analysis demonstrated 3000 red blood cells (RBCs)/mm^3^ and elevated TGs of 408 mg/dL, consistent with chylothorax. Pleural fluid cytology and flow cytometry were negative for malignant cells; however, computed tomography (CT) of the abdomen and pelvis demonstrated nonspecific retroperitoneal lymphadenopathy.

The patient was subsequently re‐hospitalized 1 month later due to persistent dyspnea and productive cough and was found to have re‐accumulation of the right‐sided chylothorax. During this admission, he was also noted to have worsening thrombocytopenia, with platelet count decreasing from 200 × 10^3^/μL to 60 × 10^3^/μL.

#### 2.1.3. Diagnostic Assessment

Further evaluation of thrombocytopenia with bone marrow biopsy revealed involvement by a kappa‐restricted B‐cell lymphoma comprising approximately 60%–70% of a markedly hypercellular bone marrow (cellularity 99%), with decreased trilineage hematopoiesis and no increase in blasts. Serum IgM level was elevated at 5207 mg/dL. Flow cytometry demonstrated a CD19+/CD20+/CD5−/CD10−/CD25− immunophenotype.

Molecular testing by next‐generation sequencing (NGS) identified the MYD88 L265P mutation with a variant allele frequency (VAF) of 2.0%. Chromosome analysis demonstrated an abnormal karyotype with deletion of Chromosome 6q. Based on the presence of LPL involving the bone marrow with associated IgM monoclonal gammopathy, the patient met diagnostic criteria for WM.

#### 2.1.4. Therapeutic Intervention

The patient was initiated on chemoimmunotherapy with bendamustine and rituximab (BR) administered in 28‐day cycles for induction therapy. Rituximab was omitted during Cycle 1 due to significantly elevated baseline IgM levels (> 4000 mg/dL) and concern for IgM flare. He experienced one additional recurrence of chylothorax after treatment initiation; however, pleural fluid re‐accumulation resolved after two cycles of chemoimmunotherapy. Due to underlying comorbidities and chemotherapy intolerance, he was subsequently transitioned to zanubrutinib.

#### 2.1.5. Follow‐Up and Outcomes

After 10 months of treatment, the patient achieved a very good partial response (VGPR) according to the International Workshop on WM (IWWM) response criteria. At follow‐up in June 2026, the patient continued to tolerate zanubrutinib 160 mg daily without significant treatment‐related cytopenias or evidence of recurrent chylothorax. He maintained an ongoing clinical response with preserved functional status and continued outpatient hematology follow‐up.

### 2.2. Patient B

#### 2.2.1. Patient Information

A 69‐year‐old male with a history of Stage IV rectal adenocarcinoma (status posttreatment and on surveillance), HTN, and chronic kidney disease (CKD) Stage 3 (attributed to recurrent acute kidney injury in the setting of prior metastatic colorectal cancer treatment, multiple chemotherapy exposures, and previous left renal infarction resulting in an atrophic left kidney with a functionally solitary right kidney) presented to UConn John Dempsey Hospital in December 2024 with progressive shortness of breath and recurrent right‐sided pleural effusion.

#### 2.2.2. Clinical Findings

**FIGURE 1:**
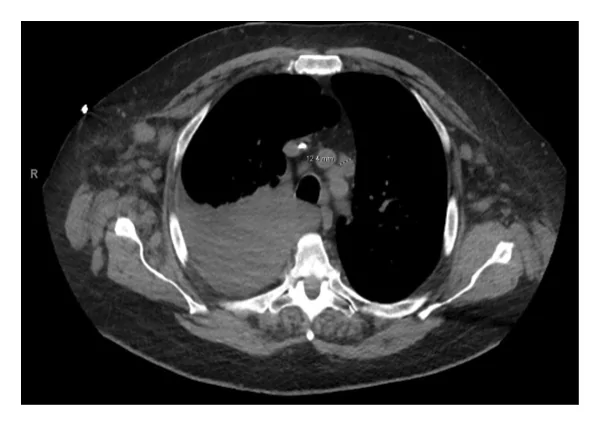
Computed tomography (CT) of the chest demonstrating a right‐sided pleural effusion associated with lymphoplasmacytic lymphoma in Patient B.

**FIGURE 2:**
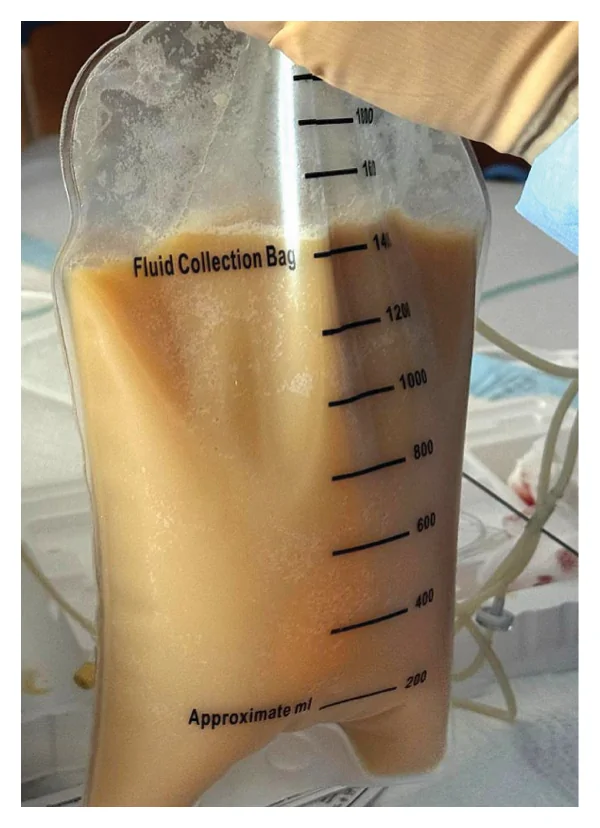
Gross appearance of chylous pleural fluid obtained during therapeutic thoracentesis in Patient B, demonstrating the characteristic milky, opaque appearance consistent with an elevated triglyceride content.

On surveillance imaging for his history of rectal cancer, the patient was found to have progressive enlargement of mesenteric, portacaval, and inguinal lymph nodes. He subsequently developed worsening shortness of breath and was found to have a large right‐sided pleural effusion (Figure 1). Pleural fluid analysis was consistent with chylothorax, with TG levels elevated at 261 mg/dL. The gross appearance of the chylous pleural fluid obtained during therapeutic thoracentesis is shown in Figure 2. The patient was also noted to have mild anemia and thrombocytopenia, prompting further hematologic evaluation.

#### 2.2.3. Diagnostic Assessment

A left inguinal lymph node excisional biopsy performed on November 1, 2024 demonstrated an atypical B‐cell lymphoid proliferation consistent with B‐cell lymphoma. Flow cytometry revealed a CD20‐positive, CD5‐negative, CD10‐negative, CD23‐positive, kappa‐restricted B‐cell population with a Ki‐67 proliferation index of approximately 20%–30%. Pleural fluid cytology was also consistent with involvement by B‐cell lymphoma. Pleural fluid flow cytometry demonstrated a clonal B‐cell population expressing CD19 and CD20, with equivocal CD5 expression. Laboratory evaluation demonstrated an IgM kappa monoclonal protein with an M‐component of 0.2 g/dL, quantitative IgM level of 297 mg/dL, elevated free kappa light chains of 68.83 mg/L, free lambda light chains of 14.75 mg/L, and kappa/lambda ratio of 4.67. Bone marrow aspirate demonstrated an atypical kappa‐restricted B‐cell population consistent with mature B‐cell lymphoma. MYD88 L265P mutation was not detected, and chromosome analysis revealed a normal karyotype. Based on the presence of LPL with IgM monoclonal gammopathy, the patient was diagnosed with WM.

#### 2.2.4. Therapeutic Intervention

Given his comorbidities, including CKD and baseline cytopenias, the patient was initially treated with single‐agent rituximab. However, he continued to experience recurrent symptomatic chylothorax requiring video‐assisted thoracoscopic surgery (VATS) pleurodesis in January 2025. Due to persistent disease‐related symptoms, bendamustine was subsequently added to rituximab therapy.

#### 2.2.5. Follow‐Up and Outcomes

Following initiation of bendamustine–rituximab (BR) therapy, the patient demonstrated clinical improvement after four treatment cycles, including improvement in respiratory symptoms and functional status. He reported improved mobility; denied fevers, chills, or night sweats; and achieved a VGPR according to IWWM response criteria after 6 months of therapy.

However, in May 2026, the patient was hospitalized with recurrent progressive dyspnea and pleuritic chest discomfort. Repeat imaging demonstrated a persistent loculated right pleural effusion with pleural calcifications, along with progressive mediastinal, hilar, axillary, mesenteric, inguinal, and retroperitoneal lymphadenopathy and new soft tissue lesions. Recurrent B‐cell lymphoma was confirmed, and second‐line therapy with rituximab and acalabrutinib was initiated.

**Patient perspective:** formal quality‐of‐life assessments were not prospectively collected. Available clinical records indicate that both patients reported subjective improvement in dyspnea and functional status following treatment initiation, prior to the subsequent events described above.

## 3. Discussion

Chylothorax associated with WM is rarely reported, and its natural history and optimal management remain incompletely characterized. Chylothorax can either be an initial manifestation of WM [4] or can occur later in the disease course after diagnosis has been established [1]. The presence of chylothorax in conjunction with lymphadenopathy should raise clinical suspicion for an underlying lymphoproliferative disorder. Given that WM predominantly involves the bone marrow, with or without peripheral lymphadenopathy or splenomegaly, bone marrow examination remains essential to confirm the diagnosis. Chylothorax in WM may arise via several overlapping mechanisms, generally classified as direct or indirect. Direct mechanisms include physical compression of the thoracic duct by enlarged lymph nodes and, in some cases, direct invasion of the duct wall by malignant lymphoplasmacytic cells, weakening its structure and causing localized leakage of lymphatic fluid [5]. Indirectly, the disease may spread to the pleura through the bloodstream, altering membrane permeability and allowing chyle to accumulate in the pleural space [6]. In addition, compression of central venous structures, particularly the superior vena cava, by bulky lymphadenopathy may elevate venous pressure and impair thoracic duct drainage [7].

It is important to note that pleural fluid analysis may not always reveal malignancy, even when chylothorax is related to an underlying lymphoproliferative disorder. As in Patient A, fluid cytology was negative despite subsequent confirmation of WM. Chylothorax can, in some cases, precede the clinical diagnosis of lymphoma [4]. Therefore, in the setting of high clinical suspicion, particularly when imaging or clinical features suggest lymphoproliferative disease, a negative pleural fluid analysis should not deter further investigation. A comprehensive workup, including imaging, lymph node assessment, and hematologic studies, is essential to avoid delayed diagnosis.

The management of chylothorax associated with lymphoma follows the general approach to malignancy‐related chylothorax. Immediate symptomatic relief should be achieved by therapeutic thoracentesis or chest tube drainage as indicated. However, symptomatic management alone is generally insufficient, and lymphoma‐directed therapy should be initiated as soon as possible. Clinicians should anticipate that responses to treatment may be gradual, and patients require close monitoring after initiation of lymphoma‐directed therapy. In the case of WM, patients may also experience urgent clinical manifestations such as hyperviscosity syndrome, which may require plasmapheresis to stabilize disease burden prior to initiation of chemoimmunotherapy, necessitating immediate pleural fluid drainage while definitive treatment is being planned.

The available literature supports adjunctive supportive measures as complements to systemic therapy. Adherence to a low‐fat diet and administration of somatostatin analogs (e.g., octreotide) may reduce the rate of chyle production and pleural fluid re‐accumulation [8]. Although surgical management is generally reserved for posttraumatic or postsurgical chylothorax, thoracic duct embolization, pleurectomy, and/or talc pleurodesis may occasionally be necessary for refractory cases despite optimal medical management or when a delayed response to medical therapy is observed [9].

The two cases described in this report highlight divergent clinical courses. In Patient A, chylothorax resolved after two cycles of BR chemoimmunotherapy, with no further pleural interventions required, and the patient achieved a durable VGPR on subsequent zanubrutinib monotherapy. In contrast, Patient B required VATS pleurodesis for refractory chylothorax despite rituximab monotherapy, ultimately requiring escalation to BR, achieving a VGPR at six months, but subsequently experiencing confirmed disease recurrence in May 2026, necessitating second‐line systemic therapy with rituximab and acalabrutinib. The more complex clinical course in Patient B may reflect multiple contributing factors, including a lower disease burden at presentation (IgM 297 mg/dL vs. 5207 mg/dL), MYD88 wild‐type status (which is associated with inferior responses to BTK inhibitor–based regimens), a normal karyotype, and a higher burden of comorbidities that limited the intensity of initial therapy.

These observations are consistent with published case reports. Otoupalova et al. described chylothorax resolution within 2 months of initiating rituximab and cyclophosphamide in a patient with WM [1], whereas Mullen et al. reported a patient requiring high‐volume therapeutic thoracentesis for more than 5 months before ultimately undergoing pleurectomy [9], highlighting the significant variability in outcomes.

The role of BTK inhibitors in the management of WM‐associated chylothorax has not been prospectively evaluated. In Patient A, chylothorax resolution had already been achieved with BR prior to transition to zanubrutinib; thus, the independent contribution of BTK inhibition to pleural disease control cannot be determined from this report. Whether direct pleural or thoracic duct involvement by lymphoma confers a different treatment response trajectory compared to indirect mechanisms also remains unclear and warrants prospective study.

The present report has several limitations. The small number of cases precludes statistical analysis or definitive conclusions regarding optimal management. Formal quality‐of‐life assessments were not prospectively collected. A median time to pleural effusion resolution could not be reliably determined given the variability in follow‐up duration and treatment trajectories. Prospective cohort studies are needed to better define the role of specific WM‐directed therapies in patients with pleural complications.

## 4. Conclusion

The association of chylothorax and WM is rare and has been scarcely reported in the literature [10]. Chylothorax should be considered in patients with unexplained nontraumatic pleural effusions and lymphadenopathy. In addition to symptomatic treatment, a comprehensive diagnostic workup, including bone marrow examination and molecular profiling, should be performed for accurate diagnosis, as early initiation of lymphoma‐directed therapy can lead to improved outcomes. The clinical courses of the two patients presented herein illustrate that therapeutic responses may vary substantially based on disease biology, molecular profile, and comorbidity burden. Prospective studies are needed to understand the clinical implications of chylothorax in WM, its mechanisms, and the impact of antilymphoma therapies on patient outcomes.

## Author Contributions

Yara Hamadah and Wan Ying Tan conceptualized the report and prepared the first draft of the manuscript. Swarup Kumar was involved in the direct clinical care of the patients. Ursula M. A. de Matos, Ritika Vankina, Kapil Meleveedu, and Swarup Kumar contributed to the acquisition and interpretation of clinical data and critically revised the manuscript for intellectual content. Yara Hamadah had full access to all of the data in this study and takes complete responsibility for the integrity and accuracy of the data.

## Funding

The authors received no specific funding for this work.

## Disclosure

All authors have read and approved the final version of the manuscript.

## Ethics Statement

This manuscript describes a report of two de‐identified clinical cases. Institutional Review Board approval was not required for this work.

## Consent

Informed consent was obtained from both patients for the publication of this case report and any accompanying de‐identified clinical images, in accordance with institutional requirements.

## Conflicts of Interest

The authors declare no conflicts of interest.

## Supporting Information

Additional supporting information can be found online in the Supporting Information section.

## Supporting information


**Supporting Information** CARE_checklist.

## Data Availability

The data supporting the findings of this report are available from the corresponding author upon reasonable request, subject to applicable patient privacy and data protection regulations.
