# Supplementary material for: Chylothorax as an Initial Presentation of Waldenström Macroglobulinemia: Report of Two Cases
Source: Case Rep Hematol. 2026 Sep 18;2026:7430484. doi: 10.1155/crh/7430484 (PMC13586783; doi:10.1155/crh/7430484)
Supplement: Supplementary file 1 [file CRH-2026-7430484-s001.docx]

**CARE Checklist — Completed**

*Manuscript: Chylothorax as an Initial Presentation of Waldenström Macroglobulinemia: Report of Two Cases*

Journal: Case Reports in Hematology | Manuscript ID: 7430484

| **Topic** | **Item** | **Checklist item description** | **Reported on page** |
| --- | --- | --- | --- |
| **Title** | | | |
| Title | 1 | The diagnosis or intervention of primary focus followed by the words "case report" | Title page |
| **Keywords** | | | |
| Key Words | 2 | 2 to 5 key words that identify diagnoses or interventions in this case report, including "case report" | Title page |
| **Abstract (no references)** | | | |
| Abstract | 3a | Introduction: What is unique about this case and what does it add to the scientific literature? | Abstract, Background |
|  | 3b | Main symptoms and/or important clinical findings | Abstract, Case Presentation |
|  | 3c | The main diagnoses, therapeutic interventions, and outcomes | Abstract, Case Presentation |
|  | 3d | Conclusion — What is the main take-away lesson(s) from this case? | Abstract, Conclusion |
| **Introduction** | | | |
| Introduction | 4 | One or two paragraphs summarizing why this case is unique (may include references) | Introduction |
| **Patient Information** | | | |
| Patient Information | 5a | De-identified patient specific information | Case Presentation, Patient Information (both patients) |
|  | 5b | Primary concerns and symptoms of the patient | Case Presentation, Clinical Findings (both patients) |
|  | 5c | Medical, family, and psycho-social history including relevant genetic information | Case Presentation, Patient Information (both patients) |
|  | 5d | Relevant past interventions with outcomes | Case Presentation, Patient B: prior rectal cancer treatment described |
| **Clinical Findings** | | | |
| Clinical Findings | 6 | Describe significant physical examination (PE) and important clinical findings | Case Presentation, Clinical Findings (both patients); Table 1 |
| **Timeline** | | | |
| Timeline | 7 | Historical and current information from this episode of care organized as a timeline | Table 2 |
| **Diagnostic Assessment** | | | |
| Diagnostic Assessment | 8a | Diagnostic testing (such as PE, laboratory testing, imaging, surveys) | Case Presentation, Diagnostic Assessment (both patients); Table 1 |
|  | 8b | Diagnostic challenges (such as access to testing, financial, or cultural) | Case Presentation: negative pleural cytology in Patient A discussed in Discussion |
|  | 8c | Diagnosis (including other diagnoses considered) | Case Presentation, Diagnostic Assessment (both patients) |
|  | 8d | Prognosis (such as staging in oncology) where applicable | Case Presentation: IWWM response criteria applied; molecular risk factors discussed in Discussion |
| **Therapeutic Intervention** | | | |
| Therapeutic Intervention | 9a | Types of therapeutic intervention (such as pharmacologic, surgical, preventive, self-care) | Case Presentation, Therapeutic Intervention (both patients) |
|  | 9b | Administration of therapeutic intervention (such as dosage, strength, duration) | Case Presentation: BR 28-day cycles; zanubrutinib 160 mg daily; VATS pleurodesis January 2025 |
|  | 9c | Changes in therapeutic intervention (with rationale) | Case Presentation, Therapeutic Intervention: Patient A transitioned to zanubrutinib (comorbidities/intolerance); Patient B escalated to BR (persistent symptoms); acalabrutinib added (disease recurrence) |
| **Follow-up and Outcomes** | | | |
| Follow-up and Outcomes | 10a | Clinician and patient-assessed outcomes (if available) | Case Presentation, Follow-up and Outcomes (both patients); Patient Perspective paragraph |
|  | 10b | Important follow-up diagnostic and other test results | Case Presentation, Follow-up and Outcomes: VGPR confirmed per IWWM criteria; May 2026 imaging findings (Patient B) |
|  | 10c | Intervention adherence and tolerability (How was this assessed?) | Case Presentation, Follow-up and Outcomes: Patient A tolerating zanubrutinib without cytopenias; Patient B denied constitutional symptoms |
|  | 10d | Adverse and unanticipated events | Case Presentation: IgM flare risk (rituximab omitted cycle 1, Patient A); disease recurrence May 2026 (Patient B) |
| **Discussion** | | | |
| Discussion | 11a | A scientific discussion of the strengths AND limitations associated with this case report | Discussion, Limitations paragraph |
|  | 11b | Discussion of the relevant medical literature with references | Discussion (all paragraphs); References 1–10 |
|  | 11c | The scientific rationale for any conclusions (including assessment of possible causes) | Discussion: mechanisms of chylothorax in WM; divergent clinical courses; MYD88 wild-type significance |
|  | 11d | The primary "take-away" lessons of this case report (without references) in a one paragraph conclusion | Conclusion |
| **Patient Perspective** | | | |
| Patient Perspective | 12 | The patient should share their perspective in one to two paragraphs on the treatment(s) they received | Case Presentation, Follow-up and Outcomes, Patient B: Patient Perspective paragraph (formal QoL assessments not collected; subjective improvement documented) |
| **Informed Consent** | | | |
| Informed Consent | 13 | Did the patient give informed consent? Please provide if requested | Yes — Declarations, Informed Consent statement |

*Note: This checklist was completed by the authors and is submitted as Supplementary File 1 in accordance with journal requirements and CARE (CAse REport) guideline recommendations.*
